# Mitochondrial genomic investigation reveals a clear association between species and genotypes of *Lucilia* and geographic origin in Australia

**DOI:** 10.1186/s13071-023-05902-1

**Published:** 2023-08-13

**Authors:** Shilpa Kapoor, Neil D. Young, Ying Ting Yang, Philip Batterham, Robin B. Gasser, Vernon M. Bowles, Clare A. Anstead, Trent Perry

**Affiliations:** 1 Bio21 Molecular Science and Biotechnology Institute, The University of Melbourne, Parkville, VIC 3010 Australia; 2 Department of Veterinary Biosciences, Faculty of Science, Melbourne Veterinary School, The University of Melbourne, Building 400, Parkville, VIC 3010 Australia

**Keywords:** Australian sheep blowfly, *Lucilia cuprina dorsalis*, *Lucilia cuprina cuprina*, *Lucilia sericata*, Mt genome, Phylogenetics

## Abstract

**Background:**

*Lucilia cuprina* and *L. sericata* (family Calliphoridae) are globally significant ectoparasites of sheep. Current literature suggests that only one of these blowfly subspecies, *L. cuprina dorsalis*, is a primary parasite causing myiasis (flystrike) in sheep in Australia. These species and subspecies are difficult to distinguish using morphological features. Hence, being able to accurately identify blowflies is critical for diagnosis and for understanding their relationships with their hosts and environment.

**Methods:**

In this study, adult blowflies (5 pools of 17 flies; *n* = 85) were collected from five locations in different states [New South Wales (NSW), Queensland (QLD), Tasmania (TAS), Victoria (VIC) and Western Australia (WA)] of Australia and their mitochondrial (mt) genomes were assembled.

**Results:**

Each mt genome assembled was ~ 15 kb in size and encoded 13 protein-coding genes, 2 ribosomal RNAs, 22 transfer RNAs and a control region. The *Lucilia* species mt genomes were conserved in structure, and the genes retained the same order and direction. The overall nucleotide composition was heavily biased towards As and Ts—77.7% of the whole genomes. Pairwise nucleotide diversity suggested divergence between *Lucilia cuprina cuprina, L. c. dorsalis* and *L. sericata*. Comparative analyses of these mt genomes with published data demonstrated that the blowflies collected from sheep farm in TAS clustered within a clade with *L. sericata*. The flies collected from an urban location in QLD were more closely related to *L. sericata* and represented the subspecies *L. c. cuprina,* whereas the flies collected from sheep farms in NSW, VIC and WA represented the subspecies* L. c. dorsalis.*

**Conclusions:**

Phylogenetic analyses of the mt genomes representing *Lucilia* from the five geographic locations in Australia supported the previously demonstrated paraphyly of *L. cuprina* with respect to *L. sericata* and revealed that *L. c. cuprina* is distinct from *L. c. dorsalis* and that *L. c. cuprina* is more closely related to *L. sericata* than *L. c. dorsalis*. The mt genomes reported here provide an important molecular resource to develop tools for species- and subspecies-level identification of *Lucilia* from different geographical regions across Australia.

**Graphical Abstract:**

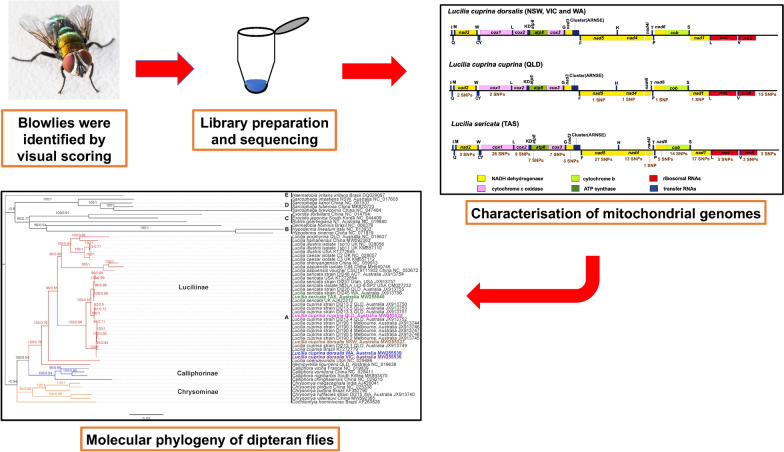

**Supplementary Information:**

The online version contains supplementary material available at 10.1186/s13071-023-05902-1.

## Background

In Australasia, blowflies of the genus *Lucilia* (Insecta, Diptera, Calliphoridae) are recognised as facultative ectoparasites of domesticated sheep but can severely affect various domestic and wild animals [1–3]. The Australian sheep blowfly, *Lucilia cuprina* (Wiedemann, 1830), causes cutaneous myiasis—a serious skin disease of sheep [4–6]. This disease results in an estimated loss of $324 million per annum to the Australian wool industry alone [7]. The common green bottle fly, *Lucilia sericata* (Meigen, 1826), is another closely related blowfly that does not cause myiasis in sheep in Australia, as it does not infest live animals as *L. cuprina* does [8]. However, *L. sericata* is the primary initiator of myiasis in sheep in Europe and is also a saprophagous species in other parts of the world [9–12]. Although both species are considered to have a cosmopolitan distribution [13, 14], *L. cuprina* is typically found more frequently in temperate subtropical areas and *L. sericata* is more prevalent in cool to temperate climes [3]. Phylogenetic studies conducted using mitochondrial (mt) gene and whole mt genome data sets have demonstrated that *L. cuprina* is paraphyletic with respect to *L. sericata* [15–17]. Populations of these two species were phylogenetically distinct, except for *L. cuprina* from Hawaii [15]. *L. cuprina* and *L*. *sericata* can hybridise, with hybrids displaying *L. cuprina*-like morphology [8]. A previous study using a combination of complementary nuclear—28S subunit ribosomal RNA (28S rRNA) and mt cytochrome *c* oxidase subunits I and II (*cox1* and *cox2*) demonstrated that these hybrids possessed *L. sericata*-type mt DNA and *L. cuprina*-type nuclear DNA [15]. Such hybrid flies have also been reported in other parts of the world, such as Asia [18], Australia [9, 17], North America [16] and South Africa [19].

Two distinct subspecies of *L. cuprina* have been described: *Lucilia cuprina cuprina* (Wiedemann, 1830) and *Lucilia cuprina dorsalis* (Robineau-Desvoidy, 1830) [8, 20, 21]. *Lucilia c. cuprina* has been identified in Australia, China, Fiji, Hawaii, India, Indonesia, Japan, Java, Malaysia, North America, South America and Timor, whereas *L. c. dorsalis* is distributed throughout Africa, Australia, India and New Zealand [8, 20, 22]. These two species as well as their subspecies are morphologically very similar and can be very challenging to identify and distinguish. *Lucilia c. cuprina* and *L. c. dorsalis* interbreed in Eastern parts of Australia [20] and produce hybrids that have overlapping morphological features complicating identification.

The purpose of this study was to gain important insights into systematic relationships of *Lucilia* species from different states of Australia using mt genomic data sets. In this study, a molecular approach was used to identify and characterise species and subspecies of *Lucilia* employing mt DNA with the aim of establishing high-quality genomic resources for these parasites. Little is known about the current distribution and genetics of these species/subspecies in Australia, which has resulted in significant bottlenecks in efforts to characterise and control these pests. Our work on the mt genomes of *Lucilia* species provides data that can be used to develop fast and reliable molecular tools that could identify and differentiate between individuals from wild populations of *Lucilia* across Australia.

## Methods

### Fly collection, DNA extraction and sequencing

Blowflies were collected from five Australian states, namely four mainland states, New South Wales (NSW), Queensland (QLD), Victoria (VIC) and Western Australia (WA), and one island state, Tasmania (TAS) (see Table 1), and were stored in RNAlater (Thermo Fisher Scientific). Flies from five unique locations were identified as *L. cuprina dorsalis, L. cuprina cuprina* or *L. sericata* based on morphological characteristics [9, 23–25]. Total genomic DNA (gDNA) was extracted from the head of each blowfly (*n* = 85) using the established approach [26, 27]. DNA quality was assessed using a 1% agarose gel and DNA quantity was measured using a Qubit Fluorometer (Invitrogen). Five genomic DNA samples, each containing equal amounts of DNA from 17 individual flies from each of the five locations in Australia, were prepared. Libraries were constructed for each of these five samples using NEBNext® Ultra™ II DNA Library Prep Kit for Illumina and paired-end sequenced using 2 × 150 cycles on an Illumina NovaSeq 6000 platform. Thus, DNA sequence data obtained from each library represented each of the five geographic locations.

**Table 1:** Australian regions and *Lucilia* species/subspecies from which isolates were derived for mitochondrial (mt) genome sequencing

Sample	Location	Latitude	Longitude
*Lucilia cuprina dorsalis*	Carwarp, VIC	36.7167°S	142.1997°E
*Lucilia cuprina dorsalis*	McMahons Reef, NSW	34.6595°S	148.4504°E
*Lucilia cuprina dorsalis*	Mount Romance, WA	34.7685°S	117.1358°E
*Lucilia cuprina cuprina*	Brisbane, QLD	27.4698°S	153.0251°E
*Lucilia sericata*	Campbell Town, TAS	41.9287°S	41.9287°S

### Mt genome assembly and annotation

The consensus mt genomes representing *Lucilia* from each of the five geographic locations in Australia were assembled (Table 1). To do this, the Trimmomatic v.0.39 program [28] was used to remove adapters, contaminants, low-quality (Phred scores < 30) and short (< 50 bp) sequencing reads. Read quality post-filtering was evaluated using the FastQC program v.0.11.9 [29], and remaining high-quality sequencing reads were used to perform a de novo assembly using NOVOPlasty v.4.2 [30]. The assembled mt genomes were annotated using the MITOS2 [31] web server with default parameters and the invertebrate mitochondrial genetic code (5) and transfer RNA (tRNA) genes were further annotated using the software ARWEN [32]. The protein-coding genes (PCGs) for each mt genome were conceptually translated based on invertebrate mitochondrial genetic code (5) and manually curated to ensure each PCG encoded a functional protein. The mt genomes were visualised using the Geneious Prime v.2019.2.3 software [33]. The mt genomes of individual *Lucilia* samples from the five individual geographic locations were drawn using OrganellarGenomeDRAW (OGDRAW) v.1.3.1 [34]. The nucleotide composition and nucleotide similarity percentages were determined using Geneious Prime v.2019.2.3 software [33]. The nucleotide composition skewness was calculated using the following formula: AT skew = (A − T)/(A + T) and GC skew = (G–C)/(G + C) [35].

### Genetic analyses

Quality-filtered FASTQ reads for each sample were mapped to the *L. c. dorsalis* mt reference genome (GenBank accession number: MW255536; VIC Australia) using Bowtie2 v2.4.5 [36]. Nucleotide polymorphisms were predicted and extracted using Annotate and Predict: Find Variations/SNPs function in Geneious Prime v.2019.2.3 software [33]. The minimum coverage and minimum variant allelic frequency was set to 5 and 0.25, respectively. Nucleotide variation was assessed within and among samples. The single-nucleotide polymorphisms (SNPs) with allelic frequency > 99% within the 17 flies for each sample were used for calculating Nucleotide diversity (π) for each PCG in a pairwise comparison using DNASP 6.12.03 [37]. Evolutionary rates including synonymous, non-synonymous substitution rates and their ratio for each species of each PCG alignment were calculated using KaKsCalculator 2.0 [38, 39].

### Relationships among *Lucilia* species/subspecies

A phylogeny was constructed using cytochrome *c* oxidase subunit I (*cox1*) sequence data for *Lucilia* species available in GenBank (NCBI; Additional file 1: Table S1). The blue blowfly *Calliphora vicina* (WA, Australia; JX913740; [17]) was used as an outgroup in the analysis. Nucleotide sequences were aligned using MAFFT v7.450 [40]. Substitution model selection for the *cox1* data set was carried out using jModeltest v2.1.10 [41, 42] and the best-fitting model was chosen using the Akaike Information Criterion. Aligned sequences were then subjected to phylogenetic analysis using Bayesian inference (BI) employing Monte Carlo Markov chain analysis in the program MrBayes v.3.2.6 [43]. Posterior probabilities (pp) were calculated using the GTR + I + G model, generating 10,000,000 trees and sampling every 200th tree until potential scale reduction factors for each parameter approached one. The final 75% of trees were used to construct a majority rule tree. Maximum likelihood (ML) analysis was performed using IQ-Tree v.1.6.12 [44] as implemented in the W-IQ-Tree web server [45]. The model of substitution was automatically selected, and an ultrafast bootstrap with 10,000 iterations was performed to investigate nodal support across the topology [46–48]. The phylogenetic tree was visualised and annotated using FigTree v1.4.4 (http://tree.bio.ed.ac.uk/software/figtree/).

To investigate the relationship of the five *Lucilia* samples and the dipteran species (Additional file 2: Table S2) available on GenBank (www.ncbi.nlm.nih.gov), a phylogenetic tree was constructed based on the combined mt gene set (13 PCGs) + 2 ribosomal RNAs (rRNAs). The phylogenetic positions of the sequenced *Lucilia* species whole mt genomes among other species of dipteran flies were examined (Additional file 2: Table S2). The nucleotide sequences of 13 PCGs and 2 rRNAs were aligned separately with MAFFT v 7.450 [40]. The buffalo fly, *Haematobia irritans irritans* (Muscidae), was used as the outgroup. After alignment, all 13 PCGs and 2 rRNAs were concatenated using Geneious Prime v.2019.2.3 [33]. Subsequently, PartitionFinder v 2.1.1 [49] was run prior to phylogenetic analysis to select the best-fit partitioning schemes and substitution models based on the Bayesian information criterion (BIC). Partitioning for the concatenated alignment was done based on the genes. Phylogenetic analysis was conducted using the program MrBayes v.3.2.6 [43]. Posterior probabilities (pp) were calculated using the GTR + I + G model, generating 10,000,000 trees and sampling every 200th tree until potential scale reduction factors for each parameter approached one. The initial 25% of sample trees were discarded as burn-in, and the others were used to construct a majority rule tree. ML analysis was performed using IQ-Tree 1.6.12 [44], as implemented in the W-IQ-Tree web server [45], using 10,000 UFBoot iterations [47, 48]. Estimation of substitution models was carried out employing ModelFinder within W-IQ-TREE [46]. The phylogenetic tree was visualised and annotated using FigTree v1.4.4 (http://tree.bio.ed.ac.uk/software/figtree/).

## Results

### Mt genome structure, organisation and composition

**Fig. 1:**
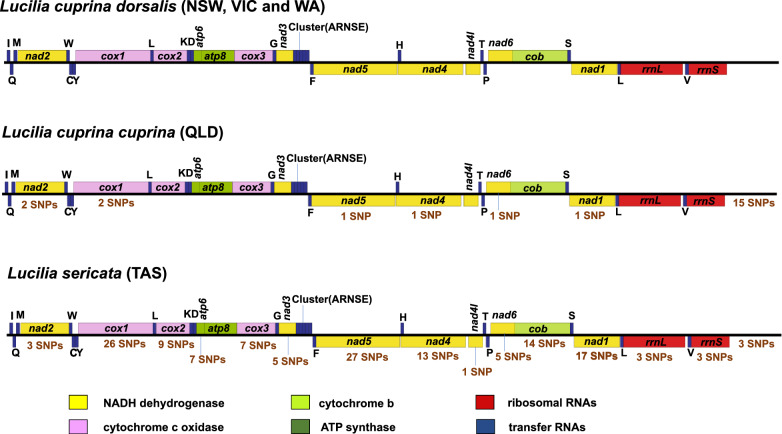
Linear maps of the circular mitochondrial (mt) genomes of *Lucilia* species/subspecies reported herein. The name of *Lucilia* species with their collection region is indicated above plots of gene order. Large bars situated on the mt genome maps indicate the position of protein‐coding genes and ribosomal RNA genes. Dark blue bars with annotated labels demarcate the positions of transfer RNA genes. Features of mt genomes are colour coded as indicated at the bottom of the figure. Protein‐coding genes are colour coded to mt complexes and the other features are coloured by type. Colours of protein‐coding, ribosomal RNA and transfer RNA genes are as follows: pink, cytochrome *c* oxidase (*cox* genes); dark green, ATP synthase (*atp* genes); yellow, NADH dehydrogenase (*nad* genes); light green, cytochrome *b* gene (*cob*); red, ribosomal RNAs and dark blue, transfer RNAs. For protein‐coding and ribosomal RNA genes, those sitting above the black line are on the positive strand, and those below the line are on the negative strand. Standard nomenclature was applied for protein-coding genes and ribosomal RNA genes, whereas for transfer RNA genes, single-letter abbreviations were used. The number of species-specific single nucleotide polymorphisms (SNPs) present in the genes were marked in brown text. Detailed annotations of the mt genomes are provided in Additional file 3: Table S3

The five mt genomes representing *Lucilia* from distinct locations in Australia were circular and 15,941 bp (*L. c dorsalis*; NSW), 15,941 bp (*L. c. dorsalis*; VIC), 15,944 bp (*L. c. dorsalis*; WA), 15,952 bp (*L. c. cuprina*; QLD) and 15,946 bp (*L. sericata;* TAS) in length/size (see Table 2). The *L. cuprina* mt genomes included here are in the size range of previously published mt genomes: *L. cuprina* strain DI213.5 (GenBank accession number: JX913753) 15,226 bp, *L. cuprina* strain DI213.2 (JX913750) 15,310 bp*, L. cuprina* strain DI190.5 (JX913748) 15,946 bp and *L. cuprina* strain DI190.1 (JX913744) 15,952 bp [17]). The lengths of previously sequenced *L. sericata* mt genomes are as follows: *L. sericata* strain DI257 (JX913757) 15,380 bp, *L. sericata* strain DI245 (JX913756) 15,214 bp, *L. sericata* strain DI220 (JX913755) 15,300 bp, *L. sericata* strain DI246 (JX913754) 15,243 bp [17], *L. sericata* (NPY120886) 15,938 bp [50] and *L. sericata* (AJ422212) 15,945 bp [51]. Based on the predicted annotation, each complete mt genome characterised herein included 37 genes consisting of 13 PCGs, 2 rRNAs [small (rrnS) and large (rrnL)], 22 tRNAs and a control region (Additional file 3: Table S3, Fig. 1). The gene organisation for the five *Lucilia* mt genomes is identical. These genomes are presumably homologous without any genome rearrangements. The longest gene was *nad5*, with a length of 1719 bp—1725 bp, and the shortest was the *atp8* gene, with a consistent length of only 165 bp. The total length of all the genes in these mt sequences represents approximately 92% of the length of the mt genomes (equivalent to 14,708 bp—14,715 bp: PCGs = 11,160 bp—11,166 bp; rRNAs = 2080 bp; tRNAs = 1468–1469 bp), and the non-coding regions were 1231–1243 bp. The start codon for most of the PCGs was ATG or ATT (Additional file 3: Table S3). For the *cox2* and *nad5* genes, incomplete stop codons were found. The most commonly observed codon was TAA as the termination codon with most initiation codons also being AT-rich.

**Table 2:** Size and skewness of the mitochondrial (mt) genomes of five *Lucilia* species/subspecies in Australia

Species/sub species	Size (bp)	A + T%	AT-skew	GC-skew
*Lucilia cuprina dorsalis* (WA)	15,944	77.70	0.016	− 0.165
*Lucilia cuprina dorsalis* (NSW)	15,941	77.70	0.016	− 0.165
*Lucilia cuprina dorsalis* (VIC)	15,941	77.70	0.016	− 0.165
*Lucilia cuprina cuprina* (QLD)	15,952	77.60	0.015	− 0.166
*Lucilia sericata* (TAS)	15,946	77.70	0.015	− 0.169
Average	15,944.8	77.68	0.016	− 0.166

The base composition of the mt sequences of all the species/subspecies was similar, with an average AT content of 77.7% (Table 2). The average AT skew was 0.016, and GC skew was –0.166 (Table 2). The frequency of nucleotide A was higher than that of T and the frequency of nucleotide C was higher than that of G. Pairwise comparison of the mt genomes revealed sequence identities of 97.8–99.9% (Table 3). The number of nucleotide differences among the five mt genomes of *Lucilia* species/subspecies ranged between 12 and 354 nucleotides. The minimum nucleotide differences (*n* = 12) were between *L. c. dorsalis* from NSW and VIC and the maximum nucleotide differences (*n* = 354) were between *L. c. dorsalis* from VIC and *L. c. cuprina* from QLD, respectively (Table 3).

**Table 3:** Nucleotide similarity percentages (number of nucleotide differences) among the five mitochondrial (mt) genomes of Australian *Lucilia* species/subspecies

	*Lucilia cuprina dorsalis* (WA)	*Lucilia cuprina dorsalis* (NSW)	*Lucilia cuprina dorsalis* (VIC)	*Lucilia cuprina cuprina* (QLD)	*Lucilia sericata* (TAS)
*Lucilia cuprina dorsalis* (WA)		99.9 (27)	99.91 (20)	97.83 (352)	97.89 (347)
*Lucilia cuprina dorsalis* (NSW)	99.9 (27)		99.93 (12)	97.84 (352)	97.91 (345)
*Lucilia cuprina dorsalis* (VIC)	99.91 (20)	99.93 (12)		97.83 (354)	97.89 (349)
*Lucilia cuprina cuprina* (QLD)	97.83 (352)	97.84 (352)	97.83 (354)		99.21 (133)
*Lucilia sericata* (TAS)	97.89 (347)	97.91 (345)	97.89 (349)	99.21 (133)	

The mt genomes are accessible in GenBank under the accession numbers MW255536–MW255540 with the NCBI BioProject accession number PRJNA419080.

### Genetic analyses

The nucleotide polymorphisms within each of the five DNA samples were recorded with respect to the *L. c. dorsalis* mt reference genome from VIC (GenBank accession number: MW255536). The number of SNPs with allelic frequency > 99% in *L. sericata* (TAS) and *L. c. cuprina* (QLD) were 198 and 78, respectively. The number of SNPs with allelic frequency < 99% in *L. sericata* (TAS), *L. c. cuprina* (QLD) and *L. c dorsalis* from NSW and WA were 26, 202, 2 and 3, respectively. There were 143 species-specific nucleotide polymorphisms in *L. sericata* (Fig. 1, Additional file 4: Table S4), with most of these markers found in the *nad5* (*n* = 27) gene followed by *cox1* (*n* = 26) and *nad1* (*n* = 17) gene (Additional file 4: Table S4). A total of 23 nucleotide polymorphisms were identified in the *L. c. cuprina* genome, which can be used to identify these blowflies from other *Lucilia* species (Fig. 1, Additional file 5: Table S5). These markers were present in the *cox1, nad1, nad2, nad4, nad5* and *nad6* genes and the non-coding region.

**Fig. 2:**
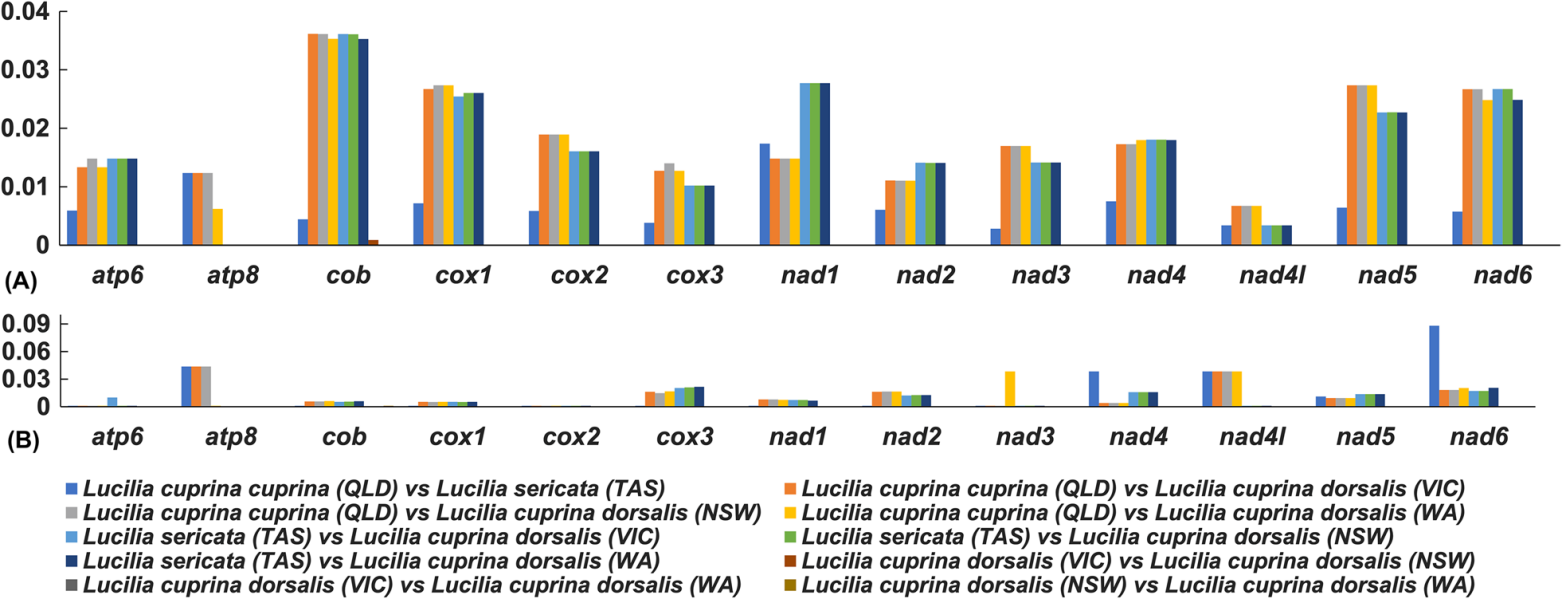
Analyses of protein-coding genes in *Lucilia* species/subspecies. **A** Nucleotide diversity (π) of individual protein-coding genes for pairs of *Lucilia* species/subspecies in this study. **B** Rates of nonsynonymous substitutions to the rate of synonymous substitutions (Ka/Ks) of individual protein-coding genes for pairs of *Lucilia* species/subspecies

Pairwise nucleotide diversity (π) analysis showed high nucleotide diversities (*π*: 0.015–0.036) for all the PCGs between *L. c. cuprina* (QLD) and *L. c. dorsalis* (VIC, NSW and WA) samples and suggested a close relationship between *L. c. dorsalis* samples (VIC, NSW and WA). Pairwise nucleotide diversity comparison for *L. c. cuprina* (QLD) and *L. c. dorsalis* (VIC, NSW and WA) showed that genetic differentiation was lowest for *nad1* (π = 0.014) and highest for *cob* (*π* = 0.036), *cox1* (*π* = 0.027), *nad5* (*π* = 0.027) and *nad6* (*π* = 0.026), respectively. Pairwise comparison of nucleotide diversity (π: 0.010–0.036) also showed differentiation between *L. sericata* (TAS) and *L. c. dorsalis* (VIC, NSW and WA) populations (Fig. 2A). The gene *atp8* showed no nucleotide diversity (*π* = 0) between *L. sericata* and *L. c. dorsalis* samples. The nucleotide diversity was highest for genes *cob* (*π* = 0.036), *cox1* (*π* = 0.026) and *nad6* (*π* = 0.026), respectively. The pairwise comparison between *L. c. cuprina* and *L. sericata* showed low nucleotide diversities (*π*: 0.002–0.017) for all the 13 PCGs. No nucleotide diversity was seen (*π* = 0) between the *L. c. dorsalis* pairwise comparisons. All the nonsynonymous to synonymous substitution (Ka/Ks) ratios were found to be < 1, suggestive of purifying selection acting on all PCGs within this group (Fig. 2B). Among all 13 PCGs, the average Ka/Ks of *nad6* is the highest (0.09) for the pairwise comparison of *L. c. cuprina* and *L. sericata*, followed by *atp8*, *nad4* and *nad4l* (Ka/Ks: 0.04 for *atp8*, 0.03 for *nad4* and *nad4l*). Overall, the *cox2* gene showed a relatively low value of Ka/Ks ratios (0.001) for all the pairwise comparisons.

### Phylogenetic analysis

**Fig. 3:**
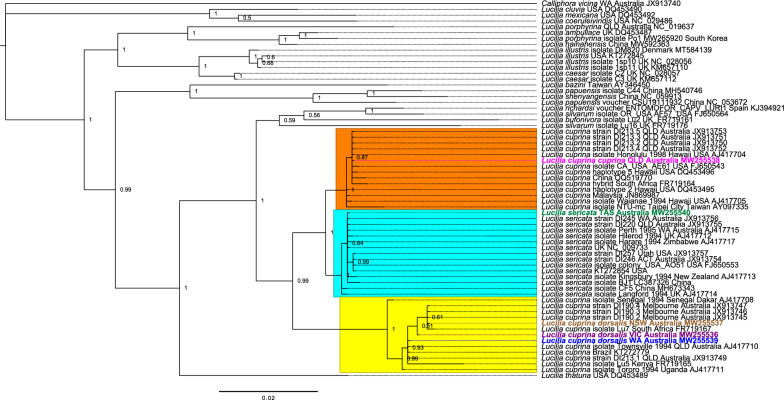
A summary of the molecular phylogeny of the *Lucilia* species/subspecies based on the cytochrome *c* oxidase subunit I *(cox1)* gene sequences. The phylogenetic tree was created using Bayesian inference (BI) implemented in MrBayes v.3.2.6. The numbers above the branches represent the posterior probabilities. Each specimen is labelled with the species name, location and GenBank accession number. Mitochondrial (mt) genomes sequenced in this study are colour coded: *Lucilia cuprina cuprina* (QLD) in pink, *Lucilia sericata* (TAS) in green, *Lucilia cuprina dorsalis* (NSW) in brown, *Lucilia cuprina dorsalis* (VIC) in purple and *Lucilia cuprina dorsalis* (WA) in blue. The *Lucilia cuprina cuprina*, *Lucilia sericata* and *Lucilia cuprina dorsalis* clades are highlighted in orange, cyan and yellow colour, respectively. The phylogram provided is presented to scale (scale bar = 0.02 estimated number of substitutions per site) with the species *Calliphora vicina* used as the outgroup

Bayesian inference (BI) and maximum likelihood (ML) analysis of the *cox1* data set revealed that all *L. sericata* samples collected from different locations around the world [JX913754 (Canberra, ACT, Australia), JX913755 (QLD, Australia), JX913756 (WA, Australia), JX913757 (Utah, USA), FJ650553 (USA), KT272854 (USA), AJ417712 (UK), AJ417713 (New Zealand), AJ422214 (UK), AJ417715 (WA, Australia), AJ417717 (Zimbabwe), MH673343 (China), LC387326 (China) and NC_009733 (UK)] clustered together in a single clade with the newly sequenced *L. sericata* (MW255540) collected from TAS, Australia (Fig. 3, Additional file 6: Figure S6). One node supporting *L. sericata* sequences from the USA (JX913757, FJ650553 and KT272854) and JX913754 (Canberra, ACT, Australia) was inferred. *Lucilia cuprina* formed two distinct clades in the phylogenetic tree. The *L. c. dorsalis* sequenced from VIC (MW255536), NSW (MW255537) and WA (MW255539) clustered in a separate clade with other *L. cuprina* sequences [JX913745 (Melbourne, Australia), JX913746 (Melbourne, Australia), JX913747 (Melbourne, Australia), JX913749 (QLD, Australia), AJ417708 (Senegal, Dakar), AJ417710 (QLD, Australia), KT272779 (Brazil), FR719165 (Kenya), FR719167 (South Africa) and AJ417711 (Uganda)]. Interestingly, *L. c. cuprina* (QLD) (MW255538) formed a clade with the *L. cuprina* flies collected from QLD, Australia (JX913750, JX913751, JX913752, and JX913753), Hawaii, USA (AJ417704, AJ417705, DQ453495 and DQ453496), California, the USA (FJ650543), Malaysia (JN869987), China (OQ519770), South Africa (FR719164) and Taipei City, Taiwan (AY097335) and shared a common ancestor with *L. sericata* (Fig. 3, Additional file 6: Fig. S6). *Lucilia illustris* sequences (KT272845; USA, NC_028056 and KM657110; UK and MT584139; Denmark) clustered within the clade with *Lucilia caesar* (NC_028057 and KM657112; UK) sequences. *Lucilia bazini* (AY346450; Taiwan [52]) grouped together with *Lucilia papuensis* (MH540746 and NC_053672) and *Lucilia shenyangensis* (NC_059913) from China with strong nodal support.

**Fig. 4:**
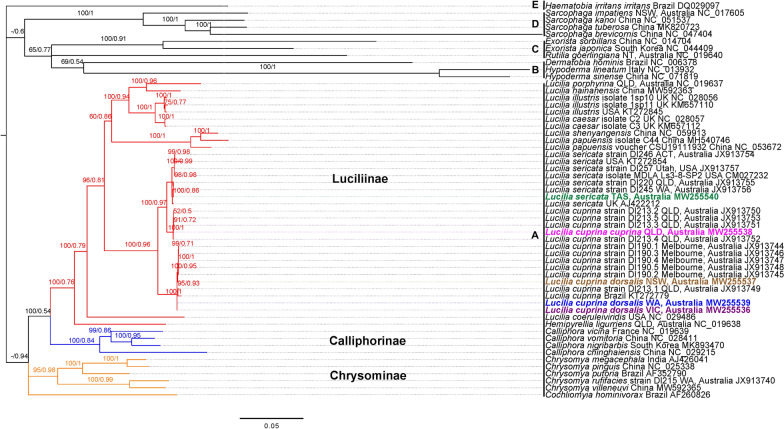
Molecular phylogeny of dipteran flies. The phylogenetic tree was created using the Bayesian inference (BI) and maximum likelihood (ML) methods. The numbers on the branches indicate bootstrap values from ML (first value) and posterior probabilities from the BI (second value) method. Hyphens "-" indicate node support values are unavailable. Each specimen is labelled with the species name, location and GenBank accession number. *Haematobia irritans irritans* from the family Muscidae was used as the outgroup. Mitochondrial (mt) genomes sequenced in this study are colour coded: *Lucilia cuprina cuprina* (QLD) in pink, *Lucilia sericata* (TAS) in green, *Lucilia cuprina dorsalis* (NSW) in brown, *Lucilia cuprina dorsalis* (VIC) in purple and *Lucilia cuprina dorsalis* (WA) in blue. To the left of the species names, family names (A: Calliphoridae, B: Oestridae, C: Tachinidae, D: Sarcophagidae and E: Muscidae) are reported. The tree branches for subfamilies Luciliinae, Calliphorinae and Chrysominae of the Calliphoridae family are colour coded in red, blue and orange, respectively. The phylogram provided is presented to scale (scale bar = 0.05 estimated number of substitutions per site)

Phylogenetic analysis of PCGs and rRNA genes based on BI and ML methods produced the same topology and showed stronger support for species and subspecies level relationships, with *L. sericata* and *L. cuprina,* separating to form species groupings with strong nodal support (Fig. 4). *Lucilia c. dorsalis* clade members collected from different locations in Australia [Melbourne, Australia (JX913744–JX913748), QLD, Australia (JX913749) and newly sequenced specimens from NSW, Australia (MW255537), VIC, Australia (MW255536) and WA, Australia (MW255539)] grouped together with *L. cuprina* from Brazil (KT272779). Flies collected from TAS (MW255540) clustered together with the *L. sericata* clade containing specimens from ACT, Australia (JX913754), the USA (JX913757, CM027232 and KT272854), UK (AJ422212), QLD, Australia (JX913755) and WA, Australia (JX913756) (Fig. 4). The subspecies *L. c. cuprina* collected from QLD, Australia (JX913750—JX913753 and MW255538) formed a sister-lineage to the *L. sericata* clade. *Lucilia c. cuprina* specimens from QLD shared a common ancestor and were more closely related to *L. sericata.* There was also good support for the sister grouping of Calliphorinae (NC_029215; China [53], MK893470; South Korea [54], NC_019639; France [17] and NC_028411; China [55]), Chrysomyinae (AJ426041; India [51], NC_025338; China, AF352790; Brazil [56], JX913740; Australia [17], MW592365; China, AF260826; Brazil [57]) with Luciliinae. The members of Sarcophagidae family (*Sarcophaga impatiens* [17], *S. kanoi* [58], *S. tubersosa* [59] and *S. brevicornis* [60]) grouped together with strong posterior probabilities and ML bootstrap values. Similarly, the members of the Tachinidae (*Exorista japonica* [61]*, E. sorbillans* [62] and *Rutilia goerlingiana* [17]) and Oestridae (*Dermatobia hominis* [63], *Hypoderma sinense* and *H. lineatum* [64]) families formed groupings with good nodal support. The molecular phylogeny based on the *cox1* gene (Fig. 3, Additional file 6: Figure S6) and 13 PCGs and 2 rRNA genes (Fig. 4) confirmed the paraphyly between *L. cuprina* and *L. sericata.*

## Discussion

*Lucilia cuprina* is an economically important pest for the sheep industry in Australia. It is difficult to accurately identify *Lucilia* species and subspecies that parasitise sheep and differentiate them from those that do not affect sheep based on morphological characteristics, such as the colour of the frontocyopeal membrane and the fore femur, position and number of hairs on the posterior slope of humeral callus and number of paravertical setulae in the central occipital area [23–25]. A further challenge is when these morphological features are not always available/accurate because of damage/degradation during collection and/or intraspecific phenotypic variation. Mt genomes can provide molecular markers for the accurate identification of poorly understood species and subspecies [65].

Blowflies were collected from single sites across five Australian states, identified using morphological characteristics and sequenced to assemble their complete mt genomes. A total of five *Lucilia* mt genomes were characterised, pairwise nucleotide diversities and the Ka/Ks ratios were calculated, and phylogenetic trees were constructed to examine the phylogenetic position of these *Lucilia* species/subspecies with respect to other Calliphoridae. All five mt genomes of *Lucilia* characterised in this study are in the size range of previously published mt genomes, with the *L. cuprina* mt genome size between 14,943 and 15,952 bp [17, 66] and the *L. sericata* mt genome size between 15,092 and 15,961 bp [17, 51, 66, 67]. The *L. sericata* genome reported here is longer (i.e. 15,946 bp) than the previously sequenced genome for *L. sericata* [17, 51, 66] and contains additional nucleotides (1–732 bp) in the non-coding regions but is shorter than *L. sericata* mt genome sequenced from North Carolina, USA [67]. The length of the *nad5* gene in the newly sequenced *L. sericata* in this study was 1725 bp, which is similar to the length of the *nad5* gene in *L. sericata* strain DI257 (GenBank accession number: JX913757), DI245 (JX913756), DI246 (JX913754) and DI220 (JX913755) [17] from Australia but 6 bp longer than the *nad5* gene (1719 bp) in *L. sericata* (AJ422212) collected from Somerset, UK [51]. In *Lucilia* species/subspecies, the *cox1* gene begins with a non-canonical start codon TCG (serine) [51, 68]. Most of the insects lack canonical (ATN) start codons at the beginning of the *cox1* gene, leading to the proposal of non-canonical start codons for the *cox1* gene [69]. For the *cox2* and *nad5* genes, incomplete stop codons were found, which has also been reported previously for members of the Calliphoridae [17, 68], and it is assumed that the termination codon is completed by polyadenylation [68, 70].

In the mt genomes, the gene order was identical to that of other *Lucilia* species sequenced to date [16, 17, 70] and is the same as that first identified in the fly *Drosophila yakuba* [71]. This mt genome arrangement is highly conserved in a wide range of different organisms with some exceptions within the class Insecta, indicating an ancestral gene order for this group [72]. The overall nucleotide composition was heavily A + T biased, accounting for 77.7% of the whole mt genome. This is typical of members of the Calliphoridae [9, 15, 73–75], including blowflies [15, 76, 77]. One of the possible biological reasons for an AT bias is due to energy efficacy trade-offs [78]. The synthesis of A and T nucleotides consumes less energy and nitrogen than G and C nucleotides [78]. The average AT skew for all five whole mt genomes was positive (0.016) and the GC skew was negative (− 0.166) indicating the bias against the use of Gs which is characteristic of the metazoan mt genome [79]. The AT and GC skews calculated for the whole mt genomes of *Lucilia* species followed the same pattern of bias as previously shown for dipteran species such as *Chrysomya chloropyga* (AF352790, AT skew: 0.020; GC skew: − 0.170) [73], *Cochliomyia hominivorax* (AF260826, AT skew: 0.034; GC skew: − 0.207) [68], *Bactrocera oleae* (AY210702 and AY210703, AT skew: 0.088; GC skew: − 0.280) [80], *Ceratitis capitata* (AJ242872, AT skew: 0.021; GC skew: − 0.185), *Drosophila melanogaster* (U37541, AT skew: 0.017; GC skew: − 0.150) [81], *D. yakuba* (X03240, AT skew: 0.005; GC skew: − 0.136) [82], *Anopheles gambiae* (L20934, AT skew: 0.032; GC skew: − 0.154) [83], *Anopheles quadrimaculatus* (L04272, AT skew: 0.041; GC skew: − 0.181) [84] and *E. sorbillans* (HQ322500, AT skew: 0.021; GC skew: − 0.171) [62].

The sequencing of mt genomes in the present study provided additional species-specific molecular markers to differentiate among *L. c. dorsalis*, *L. c. cuprina* and *L. sericata*; 143 and 23 species-specific markers were characterised for identifying *L. sericata* and *L. c. cuprina*, respectively. The mt genes *cox1*, *cox2*, *rrnS*, *nad4* and *nad4l* had been used previously for identifying members of the Calliphoridae [2, 9, 15, 16, 18, 19, 85] but a short gene sequence may not be sufficient to differentiate two species with > 95% bootstrap output [16]. Complete mt genomes provide many genetic markers that can be used to discriminate between *Lucilia* species/subspecies. To estimate the variation in 13 PCGs, nucleotide diversity was calculated. Nucleotide diversity was identified between different *Lucilia* species. The mean percentage of divergence between the *L. cuprina* and *L. sericata* clade was highest for *cob* (*π* = 2.10) followed by *cox1* (*π* = 1.63) [17], which is similar to results presented here, where nucleotide diversity for *cox1* was 0.026 between *L. cuprina* and *L. sericata*. Previously, a nucleotide diversity of *π* = 0.020 ± 0.003 had been observed among nine haplotypes amongst 24 *cox1* sequences of *L. cuprina* and *L. sericata* from South Africa [19]. *Lucilia sericata* and *L. cuprina* were clearly differentiated (by 2.8%) from each other based on the *cox1* gene sequence [18]. The pairwise comparison of nucleotide diversity based on 13 PCGs provides a better differentiation between these sister species than using a single gene.

The Ka/Ks ratio, which is used to detect selective pressure and molecular adaptation [39], was implemented to estimate the evolutionary rate in *Lucilia* species. A higher Ka/Ks ratio indicates that the gene has evolved at a faster rate than the other PCGs. The gene *nad6* (in pairwise comparison of *L. sericata* with other species/subspecies sequenced in this study) overall exhibited the highest rate of Ka/Ks ratio, which can be a result of positive selection. The gene *cox2* had the smallest Ka/Ks ratio, which indicates a strong purifying selection [77, 86]. In a similar study, the evolutionary pressure among 13 PCGs of 34 species within the Calliphoridae was investigated and showed that Ka/Ks ratio was highest for *atp8* (0.280) followed by *nad5* (0.228) and lowest for *cox1* (0.075) and *cox2* (0.090) genes, respectively [87]. The Ka/Ks ratio for the 13 PCGs of mt genomes in flesh flies (Diptera: Sarcophagidae) exhibited the highest rate (0.26) for the gene *atp8* indicating positive or relaxed selection and showed the lowest Ka/Ks ratio (0.06) for the *cox1* gene showing strong purifying selection [56]. The *cox1* barcoding gene has been widely used worldwide for many different taxonomic groups including *Lucilia* [15, 19, 85, 88]. A comparison of the *cox1* gene in our sequenced *Lucilia* species with the *cox1* sequences in the GenBank database provided insights into the evolutionary relationship between these flies but this information was limited to a few informative sites.

The phylogenetic relationship between *L. cuprina* and *L. sericata* has been analysed in the past because of their economic and welfare significance in sheep husbandry [2, 9, 15, 17, 18, 24, 89]. Phylogenetic analyses (Fig. 3, Additional file 6: Fig. S6 and Fig. 4) based on the *cox1* gene, 13 PCGs and 2 rRNAs [small (rrnS) and large (rrnL)] indicated that *L. cuprina* flies were paraphyletic to *L. sericata* with good posterior probability support and bootstrap values. This is consistent with previously demonstrated paraphyly of *L. cuprina* with respect to *L. sericata* [9, 15–19, 21, 24, 85]. The *L. sericata* collected from a sheep farm in TAS, Australia, grouped with the other specimens of *L. sericata* that had been previously shown to group together in the *L. sericata* clade [15–17, 51]. Based on the phylogenetic trees, *L. sericata* from Australia and the UK grouped together in a clade and had similar mt DNA sequences, although clear differences are reported in their ability to cause myiasis in sheep [10, 21]; *L. sericata* is the primary cause of flystrike in Europe and a saprophagous species in other parts of the world [9–12], whereas it plays a secondary role in flystrike in Australia [90]. This is consistent with the hypothesis that parasitism arose relatively recently and independently in geographically isolated populations of *Lucilia* blowflies [21, 91].

In the present study, the *L. c. dorsalis* flies collected from sheep farms in NSW, VIC and WA (Australia) clustered with the *L. cuprina* sequences obtained from GenBank (AJ417708, AJ417711, AJ417710, FR719165, FR719167, KT272779 and JX913744 to JX913749), which had been previously suggested to belong to the subspecies *L. c. dorsalis* [15, 17]. The flies which were collected from urban area of Brisbane, QLD (Australia) and identified as *L. c. cuprina* formed a clade with previously proposed subspecies *L. c. cuprina* (JX913750–JX913753, FJ650543, OQ519770, FR719164, JN869987, DQ453495, DQ453496, AJ417704, AJ417705 and AY097335) and formed a sister clade to *L. sericata*. The results of our phylogenetic analysis indicate that *L. c. cuprina* is a hybrid of *L. c. dorsalis* and *L. sericata*. The presence of *L. c. cuprina* in QLD was previously reported and it has been proposed that these flies hybridise with *L. c. dorsalis* in eastern Australia and are not known to interbreed elsewhere [17, 20]. Originally, *L. c. cuprina* was reported from Hawaii [3, 15, 21] and its presence has been reported in southeast Asia [18], South Africa [19], North America [16] and Australia [17]. *Lucilia c. cuprina* has a synanthropic behaviour, like *L. sericata*, and is concentrated in urban areas [2, 9, 17, 21, 92]. The other taxon, which is not closely related to *L. sericata*, is *L. c. dorsalis*. It is found predominantly on sheep farms and is recognised as a primary initiator of flystrike [9, 17, 20]. The present results are consistent with previous studies, showing that *L. cuprina* from QLD is closely related to *L. sericata* and likely represents the subspecies *L. c. cuprina* [9, 17]. *Lucilia* from QLD was identified as *L. c. cuprina* based on the mt genome analysis [17], and another study proposed that a blowfly specimen from Townsville, QLD, appeared to be *L. c. dorsalis* [18] based on an analysis of *cox1* sequence data [18]. In addition, it has been proposed that the *L. cuprina* from WA are *L. c. dorsalis*, and those from NSW and QLD represent *L. c. cuprina* [9]. A phylogenetic analysis including more specimens of *L. c. cuprina* flies from all Australian states and other parts of the world would be beneficial to determine the geographic distribution of these subspecies, given the tendency of the species to interbreed.

The classification of *Lucilia* species to subspecies is somewhat debatable [17, 19, 21]. The results of the phylogenetic analyses of mt DNA sequence data have indicated that *L. c. cuprina* is genetically closer to *L. sericata* than *L. c. dorsalis* [2, 9, 21]. However, the concept of two separate subspecies is not supported because the blowflies that were morphologically consistent with *L. c. cuprina* were not monophyletic in previous studies [2, 21]. Similarly, Nelson et al. [17] concluded that the two *L. cuprina* sister clades did not represent the two subspecies (*L. c. cuprina* and *L. c. dorsalis*) as the five flies collected in their study were collected at the same time and location (Petrie Terrace, Brisbane, QLD) and were morphologically consistent with *L. c. dorsalis* (JX913749 and JX913750–JX913753). Tourle et al. [19] proposed that the flies that are closely related to *L. sericata* contained nuclear pseudogenes (NUMTs); however, the hypothesis was dismissed because of the absence of false stop codons in the *cox1* sequences. A plausible explanation for the paraphyly of *L. cuprina* with respect to *L. sericata* is that there has been a hybridisation event between these two species prior to the last common ancestor of the “modern” *L. sericata* populations [17, 21, 88].

Mitochondrial DNA provides useful genetic markers or barcodes [93–95]. However, relying solely on mt DNA data for species delimitation, population genetics analyses and estimating the evolutionary history of species has some limitations [94–96]. Given that mt DNA constitutes merely a fraction (< %1) of the overall genetic material of eukaryotic cells [97], it may be insufficient for distinguishing among closely related species. This is particularly true when dealing with morphologically very similar taxa, in that relationships derived from the mt DNA data can sometimes be incongruent with those based on nuclear genomic data sets [88, 95, 98–100]. Thus, although mt DNA markers can be used as identifiers of species and subspecies [17, 68, 73], their application is constrained when addressing complex evolutionary questions, including cross-species hybridisation and introgression, where their utility may be limited [19, 88]. Therefore, future work should employ nuclear genomic data sets for detailed analyses, in order to achieve a sound understanding of the evolutionary relationships of *Lucilia* taxa and evolutionary processes [101].

## Conclusions

This study provides important insights into systematic relationships of *Lucilia* species from different states of Australia using mt genomic data sets and underpins the identification and distinction of *Lucilia* species and subspecies in the absence of reliable morphological features.

Pairwise nucleotide diversity suggests divergence among *L. c. cuprina, L. c. dorsalis* and *L. sericata*. Phylogenetic analyses reveal that *L. c. cuprina* collected from an urban location in QLD is distinct from *L. c. dorsalis* collected from sheep farms in NSW, VIC and WA and that *L. c. cuprina* is more closely related to *L. sericata* collected from TAS than *L. c. dorsalis*. In our study, the phylogenetic relationships were established solely through the analysis of mt genomes and do not reflect phylogenetic patterns observed using nuclear data.

The species-specific genetic markers are an accurate reflection of the particular genetic signatures of the species/subspecies and could aid in identifying and analysing *Lucilia* species population structure particularly in regions where their habitats overlap. Downstream analyses utilising nuclear genomic data sets will provide a deeper understanding of *Lucilia* species and subspecies.

### Supplementary Information

**Additional file 1: Table S1.** Collection details and *cox1* gene sequences for *Lucilia* species/subspecies used in the present study.

**Additional file 2: Table S2.** Collection details and mitochondrial (mt) genomic sequences for dipterans used in the present study.

**Additional file 3: Table S3.** Mitochondrial (mt) genome architecture of *Lucilia* species/subspecies collected from different locations in Australia.

**Additional file 4: Table S4.** Species-specific nucleotide polymorphisms in *Lucilia sericata* (TAS).

**Additional file 5: Table S5.** Species-specific nucleotide polymorphisms in *Lucilia cuprina cuprina* (QLD).

**Additional file 6: Figure S6.**Molecular phylogeny of the *Lucilia* species/subspecies based on the cytochrome *c* oxidase subunit I *(cox1)* gene sequences using maximum likelihood (ML) method. Each specimen is labelled with the species name, location and GenBank accession number. Mitochondrial (mt) genomes sequenced in this study are colour coded: *Lucilia cuprina cuprina* (QLD) in pink, *L. sericata* (TAS) in green, *L. cuprina dorsalis* (NSW) in brown, *L. cuprina dorsalis* (VIC) in purple and *L. cuprina dorsalis* (WA) in blue. The *L. cuprina cuprina*, *L. sericata* and *L. cuprina dorsalis* clades are highlighted in orange, cyan and yellow colour, respectively. The phylogram provided is presented to scale (scale bar = 0.02 estimated number of substitutions per site) with the species *Calliphora vicina* used as the outgroup.

## Data Availability

The datasets supporting the conclusions of this article are included within the article and its Additional files. Any additional data are available from the corresponding author upon request. The mt genomes are deposited on GenBank (accession numbers MW255536–MW255540) with the NCBI BioProject accession number PRJNA419080.

## Abbreviations

mt: Mitochondrial
NSW: New South Wales
QLD: Queensland
TAS: Tasmania
VIC: Victoria
WA: Western Australia
kb: Kilobase
bp: Base pairs
PCGs: Protein-coding genes
rRNA: Ribosomal RNA
tRNA: Transfer RNA
SNPs: Single nucleotide polymorphisms
BI: Bayesian inference
ML: Maximum likelihood
