# Supplementary material for: Mitochondrial genomic investigation reveals a clear association between species and genotypes of Lucilia and geographic origin in Australia
Source: Parasit Vectors. 2023 Aug 13;16:279. doi: 10.1186/s13071-023-05902-1 (PMC10423422; doi:10.1186/s13071-023-05902-1)
Supplement: Supplementary file 1 — Additional file 1: Table S1. Collection details and cox1 gene sequences for Lucilia species/subspecies used in the present study. [file 13071_2023_5902_MOESM1_ESM.docx]

Additional file 1: Table S1. Collection details and *cox1* gene sequences for *Lucilia* species/subspecies used in the present study.

| **Species** | **Accession number** | **Place of origin** | **Reference** |
| --- | --- | --- | --- |
| *Calliphora vicina* | JX913740 | El Questro Resort, WA, Australia (Voucher-D215) | (Nelson et al., 2012) |
| *Lucilia ampullace* | DQ453487 | UK | (Wells et al., 2007) |
| *Lucilia bazini* | AY346450 | Taiwan | (Chen at al., 2004) |
| *Lucilia bufonivora*  isolate Lu2 | FR719161 | UK | (McDonagh and Stevens, 2011) |
| *Lucilia caesar* isolate C2 | NC_028057 | UK | (Schoofs et al.)  (unpublished) |
| *Lucilia caesar* isolate C3 | KM657112 | UK | (Schoofs et al.)  (unpublished) |
| *Lucilia cluvia* | DQ453490 | USA | (Wells et al., 2007) |
| *Lucilia coeruleiviridis* | NC_029486 | USA | (Junqueira et al., 2016) |
| *Lucilia cuprina* | KT272779 | Brazil | (Junqueira et al., 2016) |
| *Lucilia cuprina* | JN869987 | Malaysia | (Chong) (unpublished) |
| *Lucilia cuprina* | OQ519770 | China | (Qu)  (unpublished) |
| *Lucilia cuprina cuprina* QLD, Australia | MW255538 | QLD, Australia | Present study |
| *Lucilia cuprina dorsalis* NSW, Australia | MW255537 | NSW, Australia | Present study |
| *Lucilia cuprina dorsalis* VIC, Australia | MW255536 | VIC, Australia | Present study |
| *Lucilia cuprina dorsalis* WA, Australia | MW255539 | WA, Australia | Present study |
| *Lucilia cuprina* haplotype 2 | DQ453495 | Hawaii, USA | (Wells et al., 2007) |
| *Lucilia cuprina* haplotype 5 | DQ453496 | Hawaii, USA | (Wells et al., 2007) |
| *Lucilia cuprina* hybrid | FR719164 | South Africa | (McDonagh and Stevens, 2011) |
| *Lucilia cuprina* isolate CA_USA_AE61 | FJ650543 | USA | (Debry et al., 2010) |
| *Lucilia cuprina* isolate Honolulu 1998 | AJ417704 | Hawaii, USA | (Stevens et al., 2002) |
| *Lucilia cuprina* isolate Lu5 | FR719165 | Kenya | (McDonagh and Stevens, 2011) |
| *Lucilia cuprina* isolate Lu7 | FR719167 | South Africa | (McDonagh and Stevens, 2011) |
| *Lucilia cuprina* isolate NTU-mc | AY097335 | Taipei City, Taiwan | (Stevens et al., 2002) |
| *Lucilia cuprina* isolate Senegal 1994 | AJ417708 | Senegal, Dakar | (Stevens et al., 2002) |
| *Lucilia cuprina* isolate Tororo 1994 | AJ417711 | Uganda | (Stevens et al., 2002) |
| *Lucilia cuprina* isolate Townsville 1994 | AJ417710 | QLD, Australia | (Stevens et al., 2002) |
| *Lucilia cuprina* isolate Waianae | AJ417705 | Hawaii, USA | (Stevens et al., 2002) |
| *Lucilia cuprina* strain DI190.2 | JX913745 | University of Melbourne colony; (P. Batterham) (Voucher - DI190.2) | (Nelson et al., 2012) |
| *Lucilia cuprina* strain DI190.3 | JX913746 | University of Melbourne colony; (P. Batterham) (Voucher - DI190.2) | (Nelson et al., 2012) |
| *Lucilia cuprina* strain DI190.4 | JX913747 | University of Melbourne colony; (P. Batterham) (Voucher - DI190.2) | (Nelson et al., 2012) |
| *Lucilia cuprina* strain DI213.1 | JX913749 | Petrie Terrace, Brisbane, QLD (Voucher-DI213.1) | (Nelson et al., 2012) |
| *Lucilia cuprina* strain DI213.2 | JX913750 | Petrie Terrace, Brisbane, QLD (Voucher-DI213.1) | (Nelson et al., 2012) |
| *Lucilia cuprina* strain DI213.3 | JX913751 | Petrie Terrace, Brisbane, QLD (Voucher-DI213.1) | (Nelson et al., 2012) |
| *Lucilia cuprina* strain DI213.4 | JX913752 | Petrie Terrace, Brisbane, QLD (Voucher-DI213.1) | (Nelson et al., 2012) |
| *Lucilia cuprina* strain DI213.5 | JX913753 | Petrie Terrace, Brisbane, QLD (Voucher-DI213.1) | (Nelson et al., 2012) |
| *Lucilia hainanensis* | MW592363 | China | (Guo nd Zhang)  (unpublished) |
| *Lucilia illustris* | KT272845 | USA | (Junqueira et al., 2016) |
| *Lucilia illustris* isolate 1sp10 | NC_028056 | UK | (Schoofs et al.) (unpublished) |
| *Lucilia illustris* isolate 1sp11 | KM657110 | UK | (Schoofs et al.)  (unpublished) |
| *Lucilia illustris* isolate DM820 | MT584139 | Denmark | (Leerhoei)  (unpublished) |
| *Lucilia mexicana* | DQ453492 | USA | (Wells et al., 2007) |
| *Lucilia papuensis* isolate C44 | MH540746 | China | (Ma and Huang)  (unpublished) |
| *Lucilia papuensis* voucher CSU19111932 | NC_053672 | China | (Guo and Ren)  (unpublished) |
| *Lucilia porphyrina* | NC_019637 | QLD, Australia | (Nelson et al., 2012) |
| *Lucilia porphyrina* isolate Po1 | MW265920 | South Korea | (Park et al.)  (unpublished) |
| *Lucilia richardsi*voucher ENTOMOFOR_CAPV_LURI1 | KJ394921 | Spain | (GilArriortua et al.)  (unpublished) |
| *Lucilia sericata* | NC_009733 | UK | (Stevens et al., 2008) |
| *Lucilia sericata* | KT272854 | USA | (Junqueira et al., 2016) |
| *Lucilia sericata* isolate BJ1 | LC387326 | China | (Xiu)  (unpublished) |
| *Lucilia sericata* isolate CF5 | MH673343 | China | (Xiu et al.)  (unpublished) |
| *Lucilia sericata* isolate colony_USA_AO51 | FJ650553 | USA | (Debry et al., 2010) |
| *Lucilia sericata* isolate Harare 1994 | AJ417717 | Zimbabwe | (Stevens et al., 2002) |
| *Lucilia sericata* isolate Hilerod 1994 | AJ417712 | UK | (Stevens et al., 2002) |
| *Lucilia sericata* isolate Kingsbury 1994 | AJ417713 | New Zealand | (Stevens et al., 2002) |
| *Lucilia sericata* isolate Langford 1994 | AJ417714 | UK | (Stevens et al., 2002) |
| *Lucilia sericata* isolate Perth 1995 | AJ417715 | WA, Australia | (Stevens et al., 2002) |
| *Lucilia sericata* strain DI220 | JX913755 | Queensland Department of Primary Industries and Fisheries Agricultural Research Institute lab strain (Voucher-DI220) | (Nelson et al., 2012) |
| *Lucilia sericata* strain DI245 | JX913756 | Perth, WA, Australia (Voucher-DI245) | (Nelson et al., 2012) |
| *Lucilia sericata* strain DI246 | JX913754 | Canberra, ACT, Australia (Voucher-DI246) | (Nelson et al., 2012) |
| *Lucilia sericata* strain DI257 | JX913757 | Brigham Young University campus, Provo, UT, USA (Voucher-DI257) | (Nelson et al., 2012) |
| *Lucilia sericata* TAS, Australia | MW255540 | TAS, Australia | Present study |
| *Lucilia shenyangensis* | NC_059913 | China | (Chen) (unpublished) |
| *Lucilia silvarum* isolate Lu16 | FR719176 | UK | (McDonagh and Stevens, 2011) |
| *Lucilia silvarum* isolate OR_USA_AF57 | FJ650564 | USA | (Debry et al., 2010) |
| *Lucilia thatuna* | DQ453489 | USA | (Wells et al., 2007) |
