# Supplementary material for: Mitochondrial genomic investigation reveals a clear association between species and genotypes of Lucilia and geographic origin in Australia
Source: Parasit Vectors. 2023 Aug 13;16:279. doi: 10.1186/s13071-023-05902-1 (PMC10423422; doi:10.1186/s13071-023-05902-1)
Supplement: Supplementary file 2 — Additional file 2: Table S2. Collection details and mitochondrial (mt) genomic sequences for dipterans used in the present study. [file 13071_2023_5902_MOESM2_ESM.docx]

Additional file 2: Table S2. Collection details and mitochondrial (mt) genomic sequences for dipterans used in the present study.

| **Species** | **Family** | **Accession number** | **Length (bp)** | **Place of origin** | **Reference** |
| --- | --- | --- | --- | --- | --- |
| *Calliphora chinghaiensis* | Calliphoridae | NC_029215 | 15269 | China | (Chen et al., 2016) |
| *Calliphora nigribarbis* | Calliphoridae | MK893470 | 16279 | South Korea | (Karagozlu et al., 2019) |
| *Calliphora vicina* | Calliphoridae | NC_019639 | 16112 | France (Voucher-DI242) | (Nelson et al., 2012) |
| *Calliphora vomitoria* | Calliphoridae | NC_028411 | 16134 | China | (Ren et al., 2016) |
| *Chrysomya megacephala* | Calliphoridae | AJ426041 | 15831 | Calicut, Kerala State, India | (Stevens et al., 2008) |
| *Chrysomya pinguis* | Calliphoridae | NC_025338 | 15838 | China | Yan et al., 2016 |
| *Chrysomya putoria* | Calliphoridae | AF352790 | 15837 | Brazil | (Junqueira et al., 2004) |
| *Chrysomya rufifacies* strain DI215 | Calliphoridae | JX913740 | 15412 | El Questro Resort, WA, Australia (Voucher-D215) | (Nelson et al., 2012) |
| *Chrysomya villeneuvi* | Calliphoridae | MW592365 | 15623 | China | (Guo and Zhang)  (unpublished) |
| *Cochliomyia hominivorax* | Calliphoridae | AF260826 | 16022 | Brazil | (Lessinger and Azeredo‐Espin, 2000) |
| *Dermatobia hominis* | Oestridae | NC_006378 | 16360 | Brazil | (Azeredo-Espin et al., 2004) |
| *Exorista japonica* | Tachinidae | NC_044409 | 17663 | South Korea | (Seo et al., 2019) |
| *Exorista sorbillans* | Tachinidae | NC_014704 | 14960 | China | (Shao et al., 2012) |
| *Haematobia irritans irritans* | Muscidae | DQ029097 | 16078 | Brazil | (Oliveira et al., 2008) |
| *Hemipyrellia ligurriens* | Calliphoridae | NC_019638 | 15938 | University of Queensland campus, St Lucia, Brisbane, Qld, Australia | (Nelson et al., 2012) |
| *Hypoderma lineatum* | Oestridae | NC_013932 | 16354 | Italy | (Weigl et al., 2010) |
| *Hypoderma sinense* | Oestridae | NC_071819 | 16296 | China | (Tang et al.)  (unpublished) |
| *Lucilia caesar* isolate C2 | Calliphoridae | NC_028057 | 15954 | UK | (Schoofs et al.)  (unpublished) |
| *Lucilia caesar* isolate C3 | Calliphoridae | KM657112 | 15957 | UK | (Schoofs et al.)  (unpublished) |
| *Lucilia coeruleiviridis* | Calliphoridae | NC_029486 | 14989 | USA | (Junqueira et al., 2016) |
| *Lucilia cuprina* | Calliphoridae | KT272779 | 14943 | Brazil | (Junqueira et al., 2016) |
| *Lucilia cuprina cuprina* QLD, Australia | Calliphoridae | MW255538 | 15952 | QLD, Australia | Present study |
| *Lucilia cuprina dorsalis* NSW, Australia | Calliphoridae | MW255537 | 15941 | NSW, Australia | Present study |
| *Lucilia cuprina dorsalis* VIC, Australia | Calliphoridae | MW255536 | 15941 | VIC, Australia | Present study |
| *Lucilia cuprina* *dorsalis* WA, Australia | Calliphoridae | MW255539 | 15944 | WA, Australia | Present study |
| *Lucilia cuprina* strain DI190.1 Melbourne, Australia | Calliphoridae | JX913744 | 15952 | University of Melbourne colony; (P. Batterham) (Voucher - DI190.1) | (Nelson et al., 2012) |
| *Lucilia cuprina* strain DI190.2 Melbourne, Australia | Calliphoridae | JX913745 | 15950 | University of Melbourne colony; (P. Batterham) (Voucher - DI190.2) | (Nelson et al., 2012) |
| *Lucilia cuprina* strain DI190.3 Melbourne, Australia | Calliphoridae | JX913746 | 15952 | University of Melbourne colony; (P. Batterham) (Voucher - DI190.3) | (Nelson et al., 2012) |
| *Lucilia cuprina* strain DI190.4 Melbourne, Australia | Calliphoridae | JX913747 | 15943 | University of Melbourne colony; (P. Batterham) (Voucher - DI190.4) | (Nelson et al., 2012) |
| *Lucilia cuprina* strain DI190.5 Melbourne, Australia | Calliphoridae | JX913748 | 15946 | University of Melbourne colony; (P. Batterham) (Voucher - DI190.5) | (Nelson et al., 2012) |
| *Lucilia cuprina* strain DI213.1 QLD, Australia | Calliphoridae | JX913749 | 15348 | Petrie Terrace, Brisbane, QLD (Voucher-DI213.1) | (Nelson et al., 2012) |
| *Lucilia cuprina* strain DI213.2 QLD, Australia | Calliphoridae | JX913750 | 15310 | Petrie Terrace, Brisbane, QLD (Voucher-DI213.2) | (Nelson et al., 2012) |
| *Lucilia cuprina* strain DI213.3 QLD, Australia | Calliphoridae | JX913751 | 15289 | Petrie Terrace, Brisbane, QLD (Voucher-DI213.3) | (Nelson et al., 2012) |
| *Lucilia cuprina* strain DI213.4 QLD, Australia | Calliphoridae | JX913752 | 15268 | Petrie Terrace, Brisbane, QLD (Voucher-DI213.4) | (Nelson et al., 2012) |
| *Lucilia cuprina* strain DI213.5 QLD, Australia | Calliphoridae | JX913753 | 15226 | Petrie Terrace, Brisbane, QLD (Voucher-DI213.5) | (Nelson et al., 2012) |
| *Lucilia hainanensis* | Calliphoridae | MW592363 | 15319 | China | (Guo nd Zhang)  (unpublished) |
| *Lucilia illustris* | Calliphoridae | KT272845 | 14875 | USA | (Junqueira et al., 2016) |
| *Lucilia illustris* isolate 1sp10 | Calliphoridae | NC_028056 | 15954 | UK | (Schoofs et al.) (unpublished) |
| *Lucilia illustris* isolate 1sp11 | Calliphoridae | KM657110 | 15956 | UK | (Schoofs et al.)  (unpublished) |
| *Lucilia papuensis* isolate C44 | Calliphoridae | MH540746 | 15884 | China | (Ma and Huang)  (unpublished) |
| *Lucilia papuensis* voucher CSU19111932 | Calliphoridae | NC_053672 | 15323 | China | (Guo and Ren)  (unpublished) |
| *Lucilia porphyrina* | Calliphoridae | NC_019637 | 15877 | University of Queensland campus, St Lucia, Brisbane, QLD, Australia (Voucher - DI211) | (Nelson et al., 2012) |
| *Lucilia sericata* | Calliphoridae | KT272854 | 15092 | USA | (Junqueira et al., 2016) |
| *Lucilia sericata* isolate MDLA Ls3-8-SP2 | Calliphoridae | CM027232 | 15961 | North Carolina, USA | (Davis et al., 2021) |
| *Lucilia sericata* strain DI220 QLD, Australia | Calliphoridae | JX913755 | 15300 | Queensland Department of Primary Industries and Fisheries Agricultural Research Institute lab strain (Voucher-DI220) | (Nelson et al., 2012) |
| *Lucilia sericata* strain DI245 WA, Australia | Calliphoridae | JX913756 | 15214 | Perth, WA, Australia (Voucher-DI245) | (Nelson et al., 2012) |
| *Lucilia sericata* strain DI246 ACT, Australia | Calliphoridae | JX913754 | 15243 | Canberra, ACT, Australia (Voucher-DI246) | (Nelson et al., 2012) |
| *Lucilia sericata* strain DI257 Utah, USA | Calliphoridae | JX913757 | 15380 | Brigham Young University campus, Provo, UT, USA (Voucher-DI257) | (Nelson et al., 2012) |
| *Lucilia sericata* TAS, Australia | Calliphoridae | MW255540 | 15946 | TAS, Australia | Present study |
| *Lucilia sericata* UK | Calliphoridae | AJ422212 | 15945 | UK | (Stevens et al., 2008) |
| *Lucilia shenyangensis* | Calliphoridae | NC_059913 | 14989 | China | (Chen)  (unpublished) |
| *Rutilia goerlingiana* | Tachinidae | NC_019640 | 15331 | Mary River Roadhouse, Burrundie, NT, Australia | (Nelson et al., 2012) |
| *Sarcophaga brevicornis* | Sacrophagidae | NC_047404 | 15152 | China | (Zhang et al., 2019) |
| *Sarcophaga impatiens* | Sacrophagidae | NC_017605 | 15169 | University of Wollongong, NSW, Australia | (Nelson et al., 2012) |
| *Sarcophaga kanoi* | Sacrophagidae | NC_051537 | 15319 | China | (She et al., 2019) |
| *Sarcophaga tuberosa* | Sacrophagidae | MK820723 | 15173 | China | (Kai et al., 2019) |
