# Supplementary material for: Mitochondrial genomic investigation reveals a clear association between species and genotypes of Lucilia and geographic origin in Australia
Source: Parasit Vectors. 2023 Aug 13;16:279. doi: 10.1186/s13071-023-05902-1 (PMC10423422; doi:10.1186/s13071-023-05902-1)
Supplement: Supplementary file 3 — Additional file 3: Table S3. Mitochondrial (mt) genome architecture of Lucilia species/subspecies collected from different locations in Australia. [file 13071_2023_5902_MOESM3_ESM.docx]

Additional file 3: Table S3. Mitochondrial (mt) genome architecture of *Lucilia* species/subspecies collected from different locations in Australia.

|  | ***L. c. dorsalis***  **(NSW)** | ***L. c. dorsalis***  **(VIC)** | ***L. c. dorsalis***  **(WA)** | ***L. c. dorsalis*** | ***L. c. cuprina***  **(QLD)** | | ***L. sericata***  **(TAS)** | | **For all mt genomes in this study** | | |
| --- | --- | --- | --- | --- | --- | --- | --- | --- | --- | --- | --- |
| **Genes** | **Location** | **Location** | **Location** | **Length (bp)** | **Location** | **Length (bp)** | **Location** | **Length (bp)** | **Transcription**  **direction** | **Start codon** | **Stop codon** |
| *rrnS* | 14087-14873 | 14087-14873 | 14088-14874 | 787 | 14091-14877 | 787 | 14088-14874 | 787 | reverse |  |  |
| *trnV(tac)* | 14017-14088 | 14017-14088 | 14018-14089 | 72 | 14021-14092 | 72 | 14018-14089 | 72 | reverse |  |  |
| *rrnL* | 12668-13960 | 12668-13960 | 12669-13961 | 1293 | 12672-13964 | 1293 | 12669-13961 | 1293 | reverse |  |  |
| *trnL1(tag)* | 12624-12688 | 12624-12688 | 12625-12689 | 65 | 12628-12692 | 65 | 12625-12689 | 65 | reverse |  |  |
| *nad1* | 11675-12613 | 11675-12613 | 11675-12613 | 939 | 11679-12617 | 939 | 11676-12614 | 939 | reverse | ATA | TAA |
| *trnS2(tga)* | 11591-11658 | 11591-11658 | 11592-11659 | 68 | 11595-11662 | 68 | 11592-11659 | 68 | forward |  |  |
| *cob* | 10456-11592 | 10456-11592 | 10456-11592 | 1137 | 10460-11596 | 1137 | 10457-11593 | 1137 | forward | ATG | TAA |
| *nad6* | 9932-10456 | 9932-10456 | 9932-10456 | 525 | 9936-10460 | 525 | 9933-10457 | 525 | forward | ATT | TAA |
| *trnP(tgg)* | 9864-9929 | 9864--9929 | 9865-9930 | 66 | 9868-9933 | 66 | 9865-9930 | 66 | reverse |  |  |
| *trnT(tgt)* | 9799-9863 | 9799-9863 | 9800-9864 | 65 | 9803-9867 | 65 | 9800-9864 | 65 | forward |  |  |
| *nad4l* | 9500-9796 | 9500-9796 | 9500-9796 | 297 | 9504-9800 | 297 | 9501-9797 | 297 | reverse | ATG | TAA |
| *nad4* | 8106-9440 | 8106-9440 | 8106-9440 | 1335 | 8110-9444 | 1335 | 8107-9441 | 1335 | reverse | ATG | TAA |
| *trnH(gtg)* | 8103-8167 | 8103-8167 | 8103-8167 | 65 | 8107-8171 | 65 | 8104-8168 | 65 | forward |  |  |
| *nad5* | 6369-8087 | 6369-8087 | 6369-8087 | 1719 | 6373-8091 | 1719 | 6370-8094 | 1725 | reverse | ATT | Incomplete |
| *trnF(gaa)* | 6301-6367 | 6301-6367 | 6302-6368 | 67 | 6305-6371 | 67 | 6302-6368 | 67 | reverse |  |  |
| *trnE(ttc)* | 6217-6282 | 6217-6282 | 6217-6282 | 66 | 6220-6286 | 67 | 6217-6283 | 67 | forward |  |  |
| *trnS1(gct)* | 6147-6214 | 6147-6214 | 6147-6214 | 68 | 6147-6214 | 68 | 6147-6214 | 68 | forward |  |  |
| *trnN(gtt)* | 6081-6146 | 6081-6146 | 6081-6146 | 66 | 6081-6146 | 66 | 6081-6146 | 66 | forward |  |  |
| *trnR(tcg)* | 6018-6080 | 6018-6080 | 6018-6080 | 63 | 6018-6080 | 63 | 6018-6080 | 63 | forward |  |  |
| *trnA(tgc)* | 5954-6018 | 5954-6018 | 5954-6018 | 65 | 5954-6018 | 65 | 5954-6018 | 65 | forward |  |  |
| *nad3* | 5598-5951 | 5598-5951 | 5598-5951 | 354 | 5598-5951 | 354 | 5598-5951 | 354 | forward | ATT | TAA |
| *trnG(tcc)* | 5533-5597 | 5533-5597 | 5533-5597 | 65 | 5533-5597 | 65 | 5533-5597 | 65 | forward |  |  |
| *cox3* | 4737-5525 | 4737-5525 | 4737-5525 | 789 | 4737-5525 | 789 | 4737-5525 | 789 | forward | ATG | TAA |
| *atp6* | 4060-4737 | 4060-4737 | 4060-4737 | 678 | 4060-4737 | 678 | 4060-4737 | 678 | forward | ATG | TAA |
| *atp8* | 3902-4066 | 3902-4066 | 3902-4066 | 165 | 3902-4066 | 165 | 3902-4066 | 165 | forward | ATT | TAA |
| *trnD(gtc)* | 3835-3901 | 3835-3901 | 3835-3901 | 67 | 3835-3901 | 67 | 3835-3901 | 67 | forward |  |  |
| *trnK(ctt)* | 3765-3835 | 3765-3835 | 3765-3835 | 71 | 3765-3835 | 71 | 3765-3835 | 71 | forward |  |  |
| *cox2* | 3077-3763 | 3077-3763 | 3077-3763 | 687 | 3077-3763 | 687 | 3077-3763 | 687 | forward | ATG | Incomplete |
| *trnL2(taa)* | 3005-3070 | 3005-3070 | 3005-3070 | 66 | 3005-3070 | 66 | 3005-3070 | 66 | forward |  |  |
| *cox1* | 1471-3009 | 1471-3009 | 1471-3009 | 1539 | 1471-3009 | 1539 | 1471-3009 | 1539 | forward | TCG | TAA |
| *trnY(gta)* | 1406-1472 | 1406-1472 | 1406-1472 | 67 | 1406-1472 | 67 | 1406-1472 | 67 | reverse |  |  |
| *trnC(gca)* | 1340-1403 | 1340-1403 | 1340-1403 | 64 | 1340-1403 | 64 | 1340-1403 | 64 | reverse |  |  |
| *trnW(tca)* | 1280-1347 | 1280-1347 | 1280-1347 | 68 | 1280-1347 | 68 | 1280-1347 | 68 | forward |  |  |
| *nad2* | 285-1280 | 285-1280 | 285-1280 | 996 | 285-1280 | 996 | 285-1280 | 996 | forward | ATT | TAA |
| *trnM(cat)* | 195-263 | 195-263 | 195-263 | 69 | 195-263 | 69 | 195-263 | 69 | forward |  |  |
| *trnQ(ttg)* | 121-189 | 121-189 | 121-189 | 69 | 121-189 | 69 | 121-189 | 69 | reverse |  |  |
| *trnI(gat)* | 58-123 | 58-123 | 58-123 | 66 | 58-123 | 66 | 58-123 | 66 | forward |  |  |
