# Supplementary material for: Mitochondrial genomic investigation reveals a clear association between species and genotypes of Lucilia and geographic origin in Australia
Source: Parasit Vectors. 2023 Aug 13;16:279. doi: 10.1186/s13071-023-05902-1 (PMC10423422; doi:10.1186/s13071-023-05902-1)
Supplement: Supplementary file 4 — Additional file 4: Table S4. Species-specific nucleotide polymorphisms in Lucilia sericata (TAS). [file 13071_2023_5902_MOESM4_ESM.docx]

Additional file 4: Table S4. Species-specific nucleotide polymorphisms in *Lucilia sericata* (TAS).

| **Position** | **Polymorphism** | **Reference** | **Variant** | **Variant Frequency** | **Gene** |
| --- | --- | --- | --- | --- | --- |
| 489 | SNP (transition) | T | C | 99.80% | *nad2* |
| 581 | SNP (transition) | G | A | 100.00% | *nad2* |
| 695 | SNP (transition) | A | G | 99.90% | *nad2* |
| 752 | SNP (transition) | A | G | 99.90% | *cox1* |
| 1524 | SNP (transition) | C | T | 100.00% | *cox1* |
| 1677 | SNP (transition) | A | G | 99.30% | *cox1* |
| 1692 | SNP (transversion) | T | A | 100.00% | *cox1* |
| 1713 | SNP (transition) | C | T | 100.00% | *cox1* |
| 1878 | SNP (transition) | C | T | 100.00% | *cox1* |
| 1908 | SNP (transition) | T | C | 100.00% | *cox1* |
| 1932 | SNP (transition) | C | T | 100.00% | *cox1* |
| 2073 | SNP (transversion) | T | A | 100.00% | *cox1* |
| 2100 | SNP (transition) | T | C | 99.10% | *cox1* |
| 2130 | SNP (transversion) | A | T | 99.90% | *cox1* |
| 2184 | SNP (transition) | T | C | 99.90% | *cox1* |
| 2253 | SNP (transversion) | A | T | 100.00% | *cox1* |
| 2283 | SNP (transition) | A | G | 99.90% | *cox1* |
| 2286 | SNP (transition) | C | T | 99.60% | *cox1* |
| 2421 | SNP (transition) | A | G | 99.30% | *cox1* |
| 2427 | SNP (transition) | C | T | 100.00% | *cox1* |
| 2563 | SNP (transition) | C | T | 99.70% | *cox1* |
| 2574 | SNP (transversion) | T | A | 99.90% | *cox1* |
| 2577 | SNP (transition) | T | C | 99.70% | *cox1* |
| 2655 | SNP (transition) | C | T | 99.70% | *cox1* |
| 2718 | SNP (transition) | C | T | 100.00% | *cox1* |
| 2739 | SNP (transition) | T | C | 100.00% | *cox1* |
| 2754 | SNP (transition) | T | C | 99.90% | *cox1* |
| 2920 | SNP (transition) | A | G | 100.00% | *cox1* |
| 2988 | SNP (transition) | A | G | 99.00% | *cox1* |
| 3172 | SNP (transition) | G | A | 100.00% | *cox2* |
| 3196 | SNP (transition) | T | C | 99.90% | *cox2* |
| 3202 | SNP (transition) | A | G | 99.80% | *cox2* |
| 3208 | SNP (transition) | G | A | 100.00% | *cox2* |
| 3310 | SNP (transition) | T | C | 99.90% | *cox2* |
| 3331 | SNP (transition) | C | T | 100.00% | *cox2* |
| 3472 | SNP (transition) | T | C | 99.90% | *cox2* |
| 3487 | SNP (transition) | C | T | 100.00% | *cox2* |
| 3496 | SNP (transition) | C | T | 100.00% | *cox2* |
| 4176 | SNP (transition) | G | A | 100.00% | *atp6* |
| 4188 | SNP (transition) | T | C | 100.00% | *atp6* |
| 4227 | SNP (transition) | G | A | 99.60% | *atp6* |
| 4324 | SNP (transition) | C | T | 99.90% | *atp6* |
| 4419 | SNP (transition) | T | C | 99.30% | *atp6* |
| 4455 | SNP (transition) | C | T | 100.00% | *atp6* |
| 4527 | SNP (transversion) | A | T | 100.00% | *atp6* |
| 4892 | SNP (transition) | T | C | 99.90% | *cox3* |
| 4901 | SNP (transversion) | A | T | 100.00% | *cox3* |
| 5073 | SNP (transition) | C | T | 99.90% | *cox3* |
| 5189 | SNP (transition) | C | T | 100.00% | *cox3* |
| 5244 | SNP (transition) | C | T | 99.80% | *cox3* |
| 5250 | SNP (transition) | G | A | 99.90% | *cox3* |
| 5480 | SNP (transition) | T | C | 99.10% | *cox3* |
| 5705 | SNP (transversion) | C | A | 100.00% | *nad3* |
| 5729 | SNP (transition) | C | T | 99.00% | *nad3* |
| 5747 | SNP (transition) | G | A | 99.90% | *nad3* |
| 5843 | SNP (transition) | G | A | 100.00% | *nad3* |
| 5901 | SNP (transition) | T | C | 99.50% | *nad3* |
| 6373 | SNP (transition) | T | C | 99.00% | *nad5* |
| 6387 | SNP (transition) | G | A | 100.00% | *nad5* |
| 6513 | SNP (transversion) | A | T | 100.00% | *nad5* |
| 6528 | SNP (transition) | G | A | 100.00% | *nad5* |
| 6669 | SNP (transversion) | T | A | 99.90% | *nad5* |
| 6696 | SNP (transversion) | C | A | 99.60% | *nad5* |
| 6759 | SNP (transversion) | T | A | 100.00% | *nad5* |
| 6762 | SNP (transversion) | T | A | 99.50% | *nad5* |
| 6774 | SNP (transition) | T | C | 99.50% | *nad5* |
| 6783 | SNP (transition) | C | T | 99.90% | *nad5* |
| 6786 | SNP (transition) | T | C | 99.30% | *nad5* |
| 6819 | SNP (transversion) | T | A | 100.00% | *nad5* |
| 6834 | SNP (transition) | T | C | 99.90% | *nad5* |
| 6840 | SNP (transition) | C | T | 100.00% | *nad5* |
| 6861 | SNP (transition) | C | T | 100.00% | *nad5* |
| 6912 | SNP (transition) | T | C | 99.80% | *nad5* |
| 7008 | SNP (transition) | G | A | 99.90% | *nad5* |
| 7023 | SNP (transition) | C | T | 99.90% | *nad5* |
| 7197 | SNP (transition) | T | C | 99.30% | *nad5* |
| 7320 | SNP (transition) | T | C | 100.00% | *nad5* |
| 7368 | SNP (transition) | T | C | 99.90% | *nad5* |
| 7371 | SNP (transition) | C | T | 100.00% | *nad5* |
| 7461 | SNP (transition) | T | C | 99.20% | *nad5* |
| 7533 | SNP (transition) | T | C | 99.80% | *nad5* |
| 7608 | SNP (transition) | T | C | 100.00% | *nad5* |
| 7665 | SNP (transversion) | T | A | 99.90% | *nad5* |
| 8044 | SNP (transition) | G | A | 99.80% | *nad5* |
| 8205 | SNP (transition) | T | C | 100.00% | *nad4* |
| 8247 | SNP (transition) | C | T | 100.00% | *nad4* |
| 8442 | SNP (transition) | C | T | 100.00% | *nad4* |
| 8502 | SNP (transversion) | A | C | 99.80% | *nad4* |
| 8640 | SNP (transition) | C | T | 100.00% | *nad4* |
| 8643 | SNP (transition) | C | T | 100.00% | *nad4* |
| 8778 | SNP (transition) | C | T | 100.00% | *nad4* |
| 8940 | SNP (transition) | T | C | 99.80% | *nad4* |
| 9016 | SNP (transition) | A | G | 99.70% | *nad4* |
| 9087 | SNP (transition) | T | C | 100.00% | *nad4* |
| 9090 | SNP (transversion) | A | C | 100.00% | *nad4* |
| 9120 | SNP (transition) | T | C | 99.90% | *nad4* |
| 9387 | SNP (transition) | T | C | 99.00% | *nad4* |
| 9584 | SNP (transition) | C | T | 99.90% | *nad4l* |
| 9946 | SNP (transition) | C | T | 99.90% | *nad6* |
| 10019 | SNP (transition) | T | C | 99.90% | *nad6* |
| 10183 | SNP (transition) | G | A | 100.00% | *nad6* |
| 10234 | SNP (transition) | C | T | 100.00% | *nad6* |
| 10240 | SNP (transition) | T | C | 99.80% | *nad6* |
| 10557 | SNP (transition) | C | T | 99.40% | *cob* |
| 10572 | SNP (transition) | C | T | 99.40% | *cob* |
| 10770 | SNP (transition) | T | C | 99.90% | *cob* |
| 10815 | SNP (transition) | T | C | 100.00% | *cob* |
| 10848 | SNP (transversion) | T | A | 100.00% | *cob* |
| 10917 | SNP (transition) | C | T | 99.60% | *cob* |
| 10924 | SNP (transition) | T | C | 100.00% | *cob* |
| 11007 | SNP (transition) | T | C | 100.00% | *cob* |
| 11070 | SNP (transition) | A | G | 99.00% | *cob* |
| 11118 | SNP (transition) | T | C | 99.90% | *cob* |
| 11179 | SNP (transition) | T | C | 99.90% | *cob* |
| 11478 | SNP (transition) | C | T | 100.00% | *cob* |
| 11527 | SNP (transition) | A | G | 99.70% | *cob* |
| 11544 | SNP (transition) | T | C | 99.90% | *cob* |
| 11699 | SNP (transition) | T | C | 99.80% | *nad1* |
| 11798 | SNP (transition) | C | T | 99.90% | *nad1* |
| 11837 | SNP (transition) | T | C | 99.20% | *nad1* |
| 11842 | SNP (transition) | T | C | 99.90% | *nad1* |
| 11852 | SNP (transversion) | A | T | 99.40% | *nad1* |
| 11858 | SNP (transition) | G | A | 99.90% | *nad1* |
| 12041 | SNP (transversion) | A | T | 99.90% | *nad1* |
| 12083 | SNP (transition) | C | T | 99.90% | *nad1* |
| 12116 | SNP (transition) | T | C | 99.80% | *nad1* |
| 12158 | SNP (transition) | C | T | 99.90% | *nad1* |
| 12200 | SNP (transition) | C | T | 100.00% | *nad1* |
| 12206 | SNP (transversion) | C | A | 99.50% | *nad1* |
| 12299 | SNP (transversion) | A | C | 99.20% | *nad1* |
| 12344 | SNP (transition) | T | C | 99.40% | *nad1* |
| 12407 | SNP (transition) | T | C | 99.90% | *nad1* |
| 12410 | SNP (transition) | C | T | 99.90% | *nad1* |
| 12592 | SNP (transition) | C | T | 99.40% | *nad1* |
| 13067 | SNP (transition) | C | T | 99.70% | *rrnL* |
| 13073 | SNP (transition) | A | G | 99.50% | *rrnL* |
| 13181 | SNP (transition) | T | C | 99.30% | *rrnL* |
| 14333 | SNP (transition) | C | T | 100.00% | *rrnS* |
| 14562 | SNP (transition) | T | C | 99.10% | *rrnS* |
| 14588 | SNP (transversion) | T | A | 99.10% | *rrnS* |
| 15393 | SNP (transition) | G | A | 100.00% |  |
| 15467 | SNP (transition) | G | A | 100.00% |  |
| 15647 | SNP (transition) | A | G | 100.00% |  |
