# Supplementary material for: Mitochondrial genomic investigation reveals a clear association between species and genotypes of Lucilia and geographic origin in Australia
Source: Parasit Vectors. 2023 Aug 13;16:279. doi: 10.1186/s13071-023-05902-1 (PMC10423422; doi:10.1186/s13071-023-05902-1)
Supplement: Supplementary file 5 — Additional file 5: Table S5. Species-specific nucleotide polymorphisms in Lucilia cuprina cuprina (QLD). [file 13071_2023_5902_MOESM5_ESM.docx]

Additional file 5: Table S5. Species-specific nucleotide polymorphisms in *Lucilia cuprina cuprina* (QLD).

| **Position** | **Polymorphism** | **Reference** | **Variant** | **Variant Frequency** | **Region/Gene** |
| --- | --- | --- | --- | --- | --- |
| 485 | SNP (transition) | A | G | 99.30% | *nad2* |
| 984 | SNP (transversion) | A | T | 99.40% | *nad2* |
| 1908 | SNP (transition) | T | C | 99.60% | *cox1* |
| 2127 | SNP (transition) | C | T | 99.40% | *cox1* |
| 7960 | SNP (transition) | C | T | 99.50% | *nad5* |
| 9192 | SNP (transversion) | T | A | 99.80% | *nad4* |
| 10334 | SNP (transition) | C | T | 99.80% | *nad6* |
| 11972 | SNP (transition) | T | C | 99.10% | *nad1* |
| 14902 | SNP (transition) | A | G | 99.00% |  |
| 14910 | SNP (transversion) | T | A | 99.30% |  |
| 14923 | SNP (transition) | C | T | 99.00% |  |
| 14958 | SNP (transition) | C | T | 99.30% |  |
| 14964 | SNP (transversion) | A | T | 99.10% |  |
| 14987 | SNP (transversion) | T | A | 99.60% |  |
| 15187 | SNP (transversion) | A | T | 99.70% |  |
| 15195 | SNP (transition) | C | T | 99.90% |  |
| 15238 | SNP (transition) | C | T | 99.90% |  |
| 15244 | SNP (transversion) | T | A | 99.80% |  |
| 15271 | SNP (transversion) | A | T | 99.90% |  |
| 15279 | SNP (transversion) | A | T | 99.90% |  |
| 15282 | SNP (transition) | T | C | 100.00% |  |
| 15316 | SNP (transition) | T | C | 99.70% |  |
| 15319 | SNP (transition) | C | T | 99.80% |  |
