# Supplementary figures and images for: Mitochondrial genomic investigation reveals a clear association between species and genotypes of Lucilia and geographic origin in Australia
Source: Parasit Vectors. 2023 Aug 13;16:279. doi: 10.1186/s13071-023-05902-1 (PMC10423422; doi:10.1186/s13071-023-05902-1)

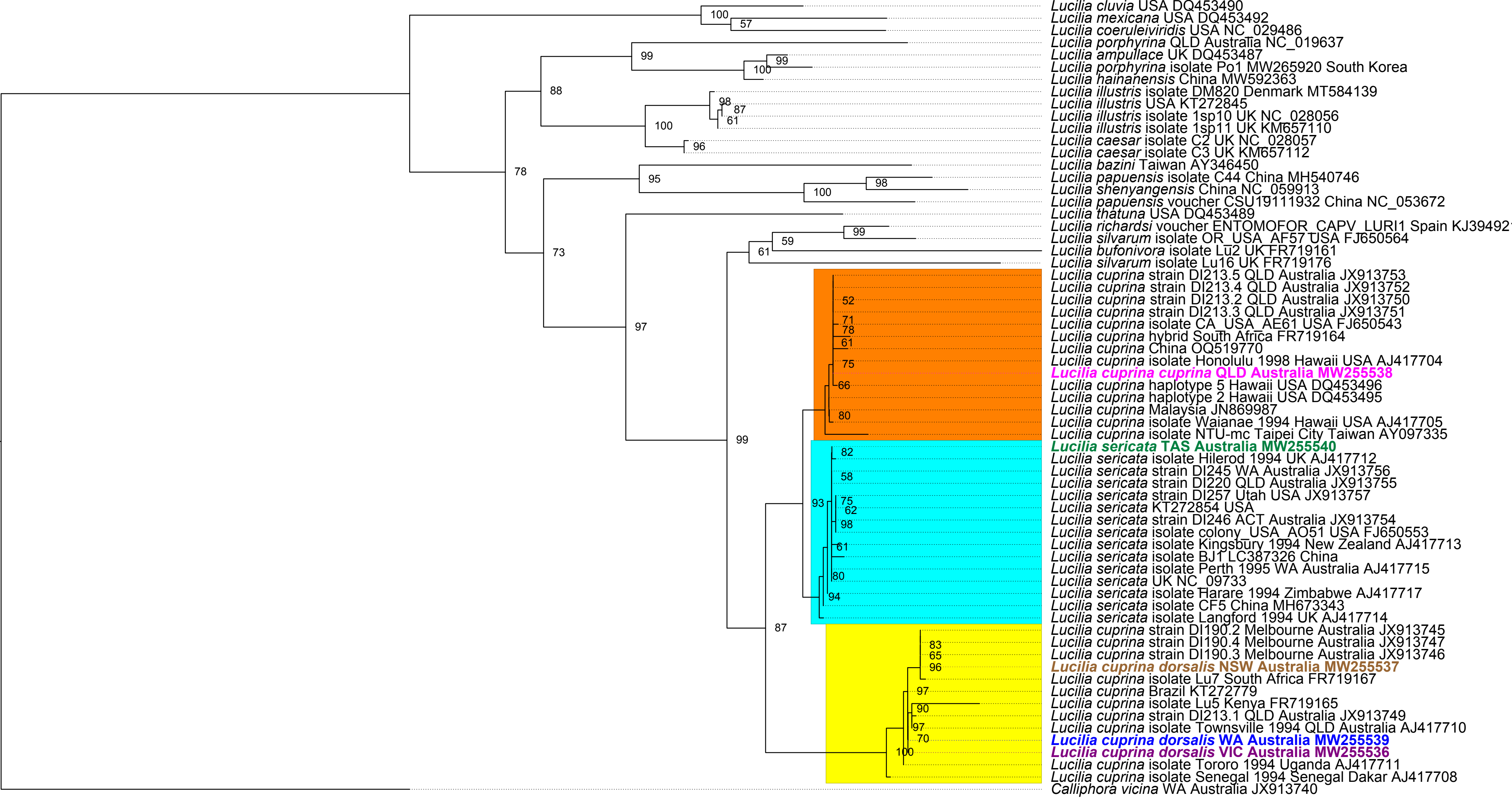

0.02

Supplement: Supplementary file 6 — Additional file 6: Figure S6.Molecular phylogeny of the Lucilia species/subspecies based on the cytochrome c oxidase subunit I (cox1) gene sequences using maximum likelihood (ML) method. Each specimen is labelled with the species name, location and GenBank accession number. Mitochondrial (mt) genomes sequenced in this study are colour coded: Lucilia cuprina cuprina (QLD) in pink, L. sericata (TAS) in green, L. cuprina dorsalis (NSW) in brown, L. cuprina dorsalis (VIC) in purple and L. cuprina dorsalis (WA) in blue. The L. cuprina cuprina, L. sericata and L. cuprina dorsalis clades are highlighted in orange, cyan and yellow colour, respectively. The phylogram provided is presented to scale (scale bar = 0.02 estimated number of substitutions per site) with the species Calliphora vicina used as the outgroup. [file 13071_2023_5902_MOESM6_ESM.pdf]
